# Topographical mapping of catecholaminergic axon innervation in the flat-mounts of the mouse atria: a quantitative analysis

**DOI:** 10.1038/s41598-023-27727-9

**Published:** 2023-04-07

**Authors:** Yuanyuan Zhang, Ariege Bizanti, Scott W. Harden, Jin Chen, Kohlton Bendowski, Donald B. Hoover, David Gozal, Kalyanam Shivkumar, Maci Heal, Susan Tappan, Zixi Jack Cheng

**Affiliations:** 1 Burnett School of Biomedical Sciences, College of Medicine, University of Central Florida, BMS Building 20, Room 230, 4110 Libra Drive, Orlando, FL 32816 USA; 2 Department of Biomedical Sciences, Quillen College of Medicine, East Tennessee State University, Johnson City, TN 37614 USA; 3 Department of Child Health and Child Health Research Institute, University of Missouri School of Medicine, Columbia, MO 65201 USA; 4 Department of Medical Pharmacology and Physiology, University of Missouri School of Medicine, Columbia, MO 65201 USA; 5 Department of Medicine, Cardiac Arrhythmia Center and Neurocardiology Research Program of Excellence, University of California, Los Angeles, CA 90095 USA; 6 MBF Bioscience, Williston, VT 05495 USA; 7 Rock Maple Science, Hinesburg, VT 05461 USA

**Keywords:** Molecular neuroscience, Neuroscience

## Abstract

The sympathetic nervous system is crucial for controlling multiple cardiac functions. However, a comprehensive, detailed neuroanatomical map of the sympathetic innervation of the heart is unavailable. Here, we used a combination of state-of-the-art techniques, including flat-mount tissue processing, immunohistochemistry for tyrosine hydroxylase (TH, a sympathetic marker), confocal microscopy and Neurolucida 360 software to trace, digitize, and quantitatively map the topographical distribution of the sympathetic postganglionic innervation in whole atria of C57Bl/6 J mice. We found that (1) 4–5 major extrinsic TH-IR nerve bundles entered the atria at the superior vena cava, right atrium (RA), left precaval vein and the root of the pulmonary veins (PVs) in the left atrium (LA). Although these bundles projected to different areas of the atria, their projection fields partially overlapped. (2) TH-IR axon and terminal density varied considerably between different sites of the atria with the greatest density of innervation near the sinoatrial node region (*P* < 0.05, n = 6). (3) TH-IR axons also innervated blood vessels and adipocytes. (4) Many principal neurons in intrinsic cardiac ganglia and small intensely fluorescent cells were also strongly TH-IR. Our work provides a comprehensive topographical map of the catecholaminergic efferent axon morphology, innervation, and distribution in the whole atria at single cell/axon/varicosity scale that may be used in future studies to create a cardiac sympathetic-brain atlas.

## Introduction

The sympathetic nervous system (SNS) plays a pivotal role in regulating cardiac functions including heart rate, contractility, and conduction velocity, which are essential for our survival^1,2^. Contrary to conventional belief, not only does the SNS play a role in the “fight or flight” integrated response, but it also regulates heart rate and contractility in both resting and non-resting conditions^3^. In fact, new emerging roles of cardiac sympathetic innervation were revealed including the regulation of cardiomyocyte size and providing a neurotrophic signal to the heart^4^. Furthermore, any disturbance of the SNS functions, including structural remodeling and overactivity, may promote progression of various cardiovascular diseases^5^. Although the functional roles of the SNS have been well established, a comprehensive organization map of the sympathetic postganglionic innervation of the atria remains insufficiently delineated. In addition, the regional density of the sympathetic innervation of the heart has yet to be quantified. There are numerous unanswered questions related to the detailed anatomy of the heart's sympathetic nervous system and how it is modified by disease states, such as atrial fibrillation, arrhythmia, and heart failure^6^. For example, a complete understanding of the morphology and morphometry to explain the complexity of sympatho-cardiac communication and the differential regional distribution of the atrial nerve plexus remains to be elucidated^1,7,8^.

Previous studies investigated the structure and function of sympathetic neurons and axons in different species^9–14^ using sectioned heart preparations or focused only on specific regions of the atria, which disrupted the continuity of axons and terminals, preventing large scale morphological characterization of these structures. Great effort has been made to better characterize the intrinsic cardiac plexus in the whole-mount mouse heart, which increased our knowledge on the distribution of noradrenergic innervation of the mouse heart^15,16^. Nevertheless, the complete fine details of TH-IR axon terminals and varicosities were not fully visualized in the whole-mount. Additionally, thick regions of the auricle and other structures were partially or completely removed. These structures include right cranial vein (RCV), left cranial vein (LCV), and caudal vein (CV)^15,17^ which we refer to in this study and our previous work as superior vena cava (SVC), left precaval vein (LPCV), and inferior vena cava (IVC)^14,18,19^; respectively. Moreover, the topology of sympathetic neurons and their local communication with the heart, which influence cardiac functions were characterized^4,20^. In those studies, it was shown that sympathetic neurons directly communicate with cardiomyocytes in the ventricles and the density of innervation correlates with the size of cardiomyocytes, all of which emphasize the need to determine the differential regional innervation of the heart. Recently, researchers were able to generate two- and three-dimensional reconstructions of the sympathetic innervation of the myocardium. However, these studies provided imaging from only a few myocardial sections and a small segment of the heart^21^. Alternatively, they revealed the big bundles without a clear visualization of the fine axons and terminals or cardiac targets^22^. Both studies used tyrosine hydroxylase (TH) as a sympathetic marker and showed that sympathetic nerves and intrinsic cardiac ganglia were distributed in both atria of the heart, predominantly near the SAN, AVN and around the junction of left and right atria^23,24^.

Despite substantial advances in knowledge on the anatomy and physiology of cardiac nerves that contribute to therapeutic responses, there are still many gaps that need to be filled as neuromodulation treatments move away from pharmaceuticals and non-specific treatments to more guided and specific therapeutic targets for cardiovascular diseases. To facilitate these transitions, the architecture of cardiac sympathetic nerves needs to be carefully and precisely determined. More studies are needed to determine the structural organization of the sympathetic postganglionic innervation of whole-mount preparations of the heart (atria and ventricles) to improve understanding of sympathetic control of the heart.

Previously, we have determined the distribution and morphology of parasympathetic afferent and efferent axons in the atria in wild-type rat and mouse preparations^25–29^ as well as in disease models (e.g., aging, sleep apnea, and diabetes)^26,30,31^. Collectively, the present work provides a comprehensive topographical map of the catecholaminergic efferent axon distribution, density, and morphology of the atria at the single cell/axon/varicosity resolution. This anatomical map will provide a foundation for future functional studies of sympathetic control of the heart and its remodeling in pathological conditions.

## Materials and methods

### Animals and ethical statement

All procedures were approved by the University of Central Florida Animal Care and Use Committee (HURON PROTO202000150) and strictly followed the guidelines established by the National Institutes of Health (NIH) and the ARRIVE 2.0 guidelines. This study was performed on healthy male C57Bl/6 J mice (RRID: IMSR_JAX000664, The Jackson Laboratory, Bar Harbor, ME) (n = 20, age 2–3 months, weighing 20–30 g). Mice were housed in a plastic cage (n = 5/cage) with sawdust bedding (changed three times a week) in a room with controlled environmental conditions of humidity and temperature in which light/dark cycles were set to 12/12 h (6:00 AM to 6:00 PM light cycle) and provided food and water ad libitum. Mice were divided into 3 groups. Connected atria TH-IR axon innervation mapping group (n = 5) were used to show topographical innervation and reconstruction of nerves. Quantification analysis of separate right and left atria group (n = 6) were used to perform regional density analysis. Control group (n = 4) were used to ensure there were not any nonspecific labeling and that labelled structures represent neuronal and axonal structures. This was performed by omitting the primary antibody (n = 1) or omitting the secondary antibody (n = 1) and labelling with PGP9.5 (3). Additional animals were used to counter-stain neurons with Fluorogold (n = 4). All efforts were made to minimize the number of mice and their suffering.

### Tissue preparation

Mice were deeply anesthetized with isoflurane (4%) induction in an anesthetic chamber. Absence of the hind paw pinch withdrawal reflex was used as an indicator of sufficient depth of anesthesia. Mice were injected with 0.2 mL heparin into the left ventricle followed by a cut to the inferior vena cava to drain the blood. After 2 min, a needle was inserted into the left ventricle and the mice were perfused with 0.9% saline at 38–40 °C for 5 min, followed by fixation with 4% paraformaldehyde. Hearts along with the lungs and trachea were removed from the chest and postfixed overnight in 4% paraformaldehyde at 4 °C. The heart was placed and pinned into a dissecting dish lined with Sylgard and containing PBS (0.1 M, pH = 7.4), and the specimen was further dissected using a Leica Stereo microscope as described previously^25,27,28,32,33^. To reveal the intact network of sympathetic postganglionic atrial innervation, we removed the heart from the surrounding tissues (lungs, aortic arch and trachea). Then, the atria (both right and left atrium connected at the interatrial septum on the ventral side) were separated from the ventricles (n = 5). The whole atria were processed as a montage of several hundred (~ 260) maximal projections of image stacks. To gain more insight into TH-IR axon innervation and regional density, the right and left atria (RA and LA) were separated. The auricles were cut along the boundary into two halves. The part of the auricle facing more exteriorly and connected to the big vessels is referred to in this study as the outer auricle and the other half is referred to as the inner auricle. Then, flat-mounts were scanned using the confocal microscope at higher magnification (40X oil lens). The separation of the atria was necessary to avoid areas of overlap between RA and LA. Montages of the maximal projections of the right and left atria were prepared (n = 6/group). A detailed experimental protocol^34^ is available through Protocol.io: 10.17504/protocols.io.n92ldzbmxv5b/v2.

### Immunohistochemistry (IHC)

Tissue processing and immunolabeling were performed as described previously^32^. Following dissection, the tissues were washed 6 × 5 min in 0.1 M PBS (pH = 7.4), then immersed for 48 h in a blocking reagent (2% bovine serum albumin, 10% normal donkey serum, 2% Triton X-100, 0.08% NaN_3_ in 0.1 M PBS, pH = 7.4) to reduce nonspecific binding of the primary antibody and to promote increased antibody penetration. Primary antibodies (1:100) were added to the primary solution (2% bovine serum albumin, 4% normal donkey serum, 0.5% Triton X-100, 0.08% NaN_3_ in 0.1 M PBS, pH = 7.4) and incubated for 48 h. Unbound primary antibodies were removed by 6 × 5 min tissue washes in PBST (0.5% Triton X-100 in 0.1 M PBS, pH = 7.4). Secondary antibodies (1:50 in PBST) were then applied for 24 h. Unbound secondary antibodies were removed by 6 × 5 min tissue washes in PBS. Negative control tests (in which primary antibodies were omitted) were also performed, and these preparations presented no labeling, confirming that nonspecific binding of secondary antibodies did not occur. Lastly, we verified the accuracy of our TH labeling by using PGP 9.5 (ubiquitin carboxyl-terminal hydrolase-1), a general neuronal marker that visualizes different populations and subtypes of nerves. A list of the antibodies used in this study is summarized in Table 1.

**Table 1:** Antibodies used in this study.

Antibody	Concentration	Host	Company	Catalog	RRID
Anti-TH	1:100	Rabbit	Pel-Freeze	Cat# P40101-0	AB_461064
Anti-TH	1:100	Sheep	Millipore	AB1542	AB_90755
Anti- PGP9.5	1:100	Rabbit	Abcam	ab108986	AB_10891773
Alexa Fluor 594 anti-sheep	1:50	Donkey	Invitrogen	A-11016	AB_2534083
Alexa Fluor 488 anti-rabbit	1:50	Donkey	Invitrogen	A21206	AB_2535792

Flat-mounts were placed on a microscope slide with their dorsal side against the glass, coverslipped, crushed for 2 days with lead weights, and air-dried under a fume hood for 1 day. Slides were dehydrated by immersion for 2 min in each of 4 ascending concentrations of ethanol (75, 95, 100 and 100%), followed by 2 × 10 min washes in xylene. Slides were then covered with coverslips and DEPEX mounting medium (Electron Microscopy Sciences #13514) and allowed to dry overnight.

### Fluoro-Gold (FG) counterstaining

To evaluate the location of immunolabeled structures relative to cardiac ganglia, FG was used to to counterstain neurons in four additional animals. Fluoro-Gold (0.3 mL of 3 mg/mL per mouse; Fluorochrome, LLC, FG 50 mg) was injected (i.p.) to counter stain neurons in the peripheral ganglia. Mice were perfused 3–5 days after FG injection and the hearts were removed and dual labeled with TH.

### Image acquisition

The Nikon 80i fluorescence microscope (Lens: 20X and 40X) was first used to survey the TH labeling in the whole flat-mounts of the atria. Then, a Leica TCS SP5 laser scanning confocal microscope (Lens: 20X and 40X oil) was used to acquire images and assemble image montages of whole connected atria, including left atrium and right atrium flat-mounts. An argon-krypton laser (excitation 488 nm) was used to image TH-IR axons, a helium-neon (HeNe) laser (excitation 543 nm) was used to image PGP9.5-IR axons, and UV laser was used to detect FG or background autofluorescence of the tissues. The connected atria were scanned using a 20X oil immersion objective lens (Z-step 1.5 μm), to produce approximately 400 confocal image stacks per montage. The confocal projection images of these stacks were used to assemble montages of whole atria flat-mounts using either Mosaic J or photoshop. To better visualize the topographical distribution and morphology of TH-IR innervation in the atria, the separate whole left atrium and right atrium and regions of interest were scanned at high magnification (40X oil immersion objective lens, Zoom X1 or X 1.5, Z-step 1.5 μm). The higher magnification resulted in approximately 800 frames for each atrium. We were able to overcome the thickness of the flat-mount whole atria with our optimized tissue processing techniques and flattening of the tissue which allowed us to visualize fine details of TH-IR axon innervation. We also used a Zeiss M2 Imager microscope with an autostage (20X NA 0.8) to scan the samples which produced images with high quality that were comparable to the images obtained with confocal microscopy (20X objective lens). This approach will make future methodology less laborious and more efficient.

Tracing of TH-IR axons was performed using Neurolucida 360 (MBF Bioscience). Additionally, Neurolucida Explorer (MBF Bioscience), an analytical software built within Neurolucida 360, was used to perform morphometric analysis on traced axon reconstructions. Branched structure analysis was performed, and parameters (number of trees, nodes, terminals, total length and surface area) were selected for all connected atria tracings (n = 6).

### Density and size quantification

**Figure 1:**
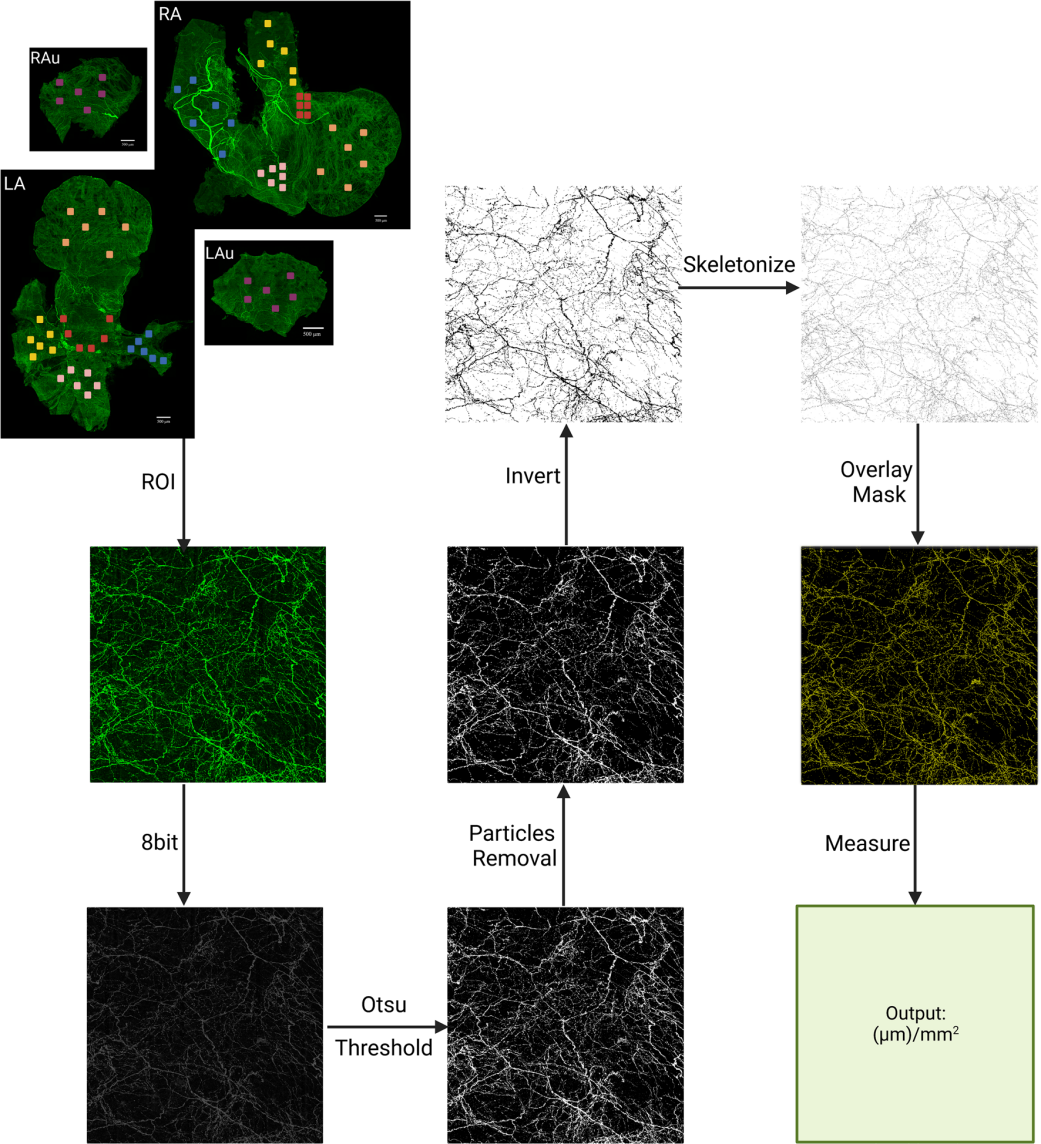
Pipeline for calculation of TH-IR axon density. Six frames within each region of interest (ROI) were selected, converted to grayscale, thresholded using Otsu algorithm, skeletonized and the signal pixels were measured in each ROI to represent total length of axons travelled in each ROI.

To quantitate the regional density of TH-IR fibers in the atria, we segregated images into specific regions of interest (ROIs): SAN, AVN, SVC, IVC, right outer and inner auricle, LA-PV junction, left PV, middle PV, right PV, left outer and inner auricle using Fiji^35^. The steps of density quantification were as follows (Fig. 1): (1) Subtracted the background with radius of 80 pixels to reduce noise and enhance contrast. (2) Applied particle removal to remove small debris. (3) Applied a binary threshold (Otsu method)^36^ to isolate immunoreactive structures. (4) Quantified the signal above the threshold. (5) Averaged the signal of different ROIs windows using six counting frames. (6) Ran the Shapiro–Wilk normality test. Axontracer algorithm was used to trace and confirm axon quantification^37^. Axon density was represented as total axon length per ROI. Total axon length in pixels was converted to μm using appropriate conversion factors. Statistical significance of the difference between the means was performed using one-way ANOVA and Tukey’s HSD (Honestly Significant Difference). Data are expressed as means + / − SEM. Significance is accepted at *P* < 0.05.

Heatmaps were created after applying a modified version of the freely available open-source automated software algorithm that trace and quantify axons (Axon tracer plugin, ImageJ)^37^. The percentage of TH-IR neurons was counted using all single optical sections of different ICG image stacks.

## Results

### Topographical projections of TH-IR axons in the flat-mount of the whole left and right atria (connected): Neurolucida tracing and digitization

**Figure 2:**
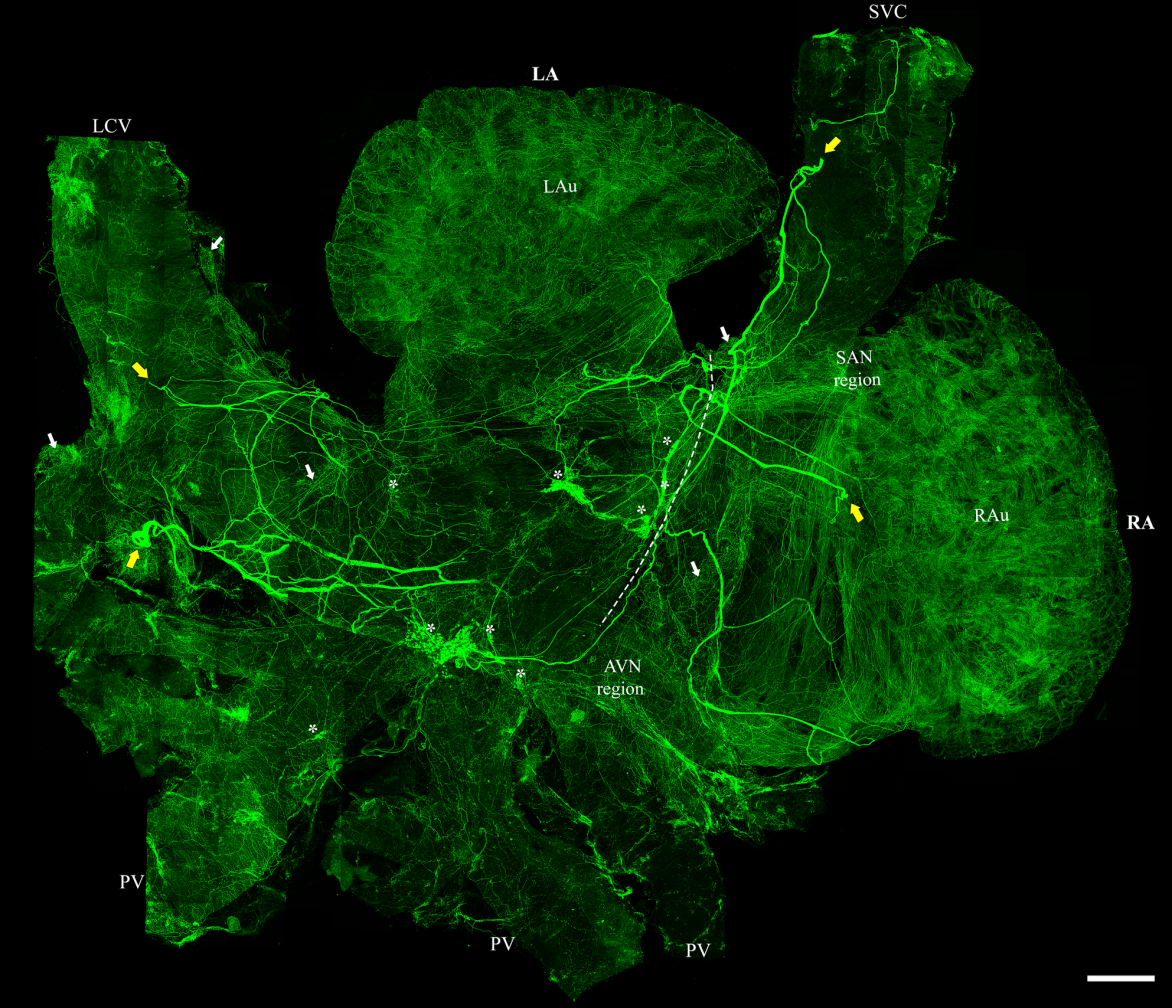
Distribution of TH-IR axons in the flat-mount of the whole atria (RA and LA connected). A montage of 260 maximal intensity projection images showed several large TH-IR bundles that entered the atria and bifurcated into small bundles that innervated the entire connected RA and LA. TH-IR neurons in the ICG were more densely distributed in the LA, junction of LA and RA, entrance of LA to PV. Intrinsic cardiac ganglia (asterisks), Fat (white arrows), junction of LA and RA (dotted line), entrance points (yellow arrows). *LA* left atrium, *RA* right atrium, *PV* pulmonary vein, *SVC* superior vena cava, *LPCV* left precaval vein, *SAN* sinoatrial node region, *AVN* atrioventricular node region. Scale bar: 500 μm.

**Figure 3:**
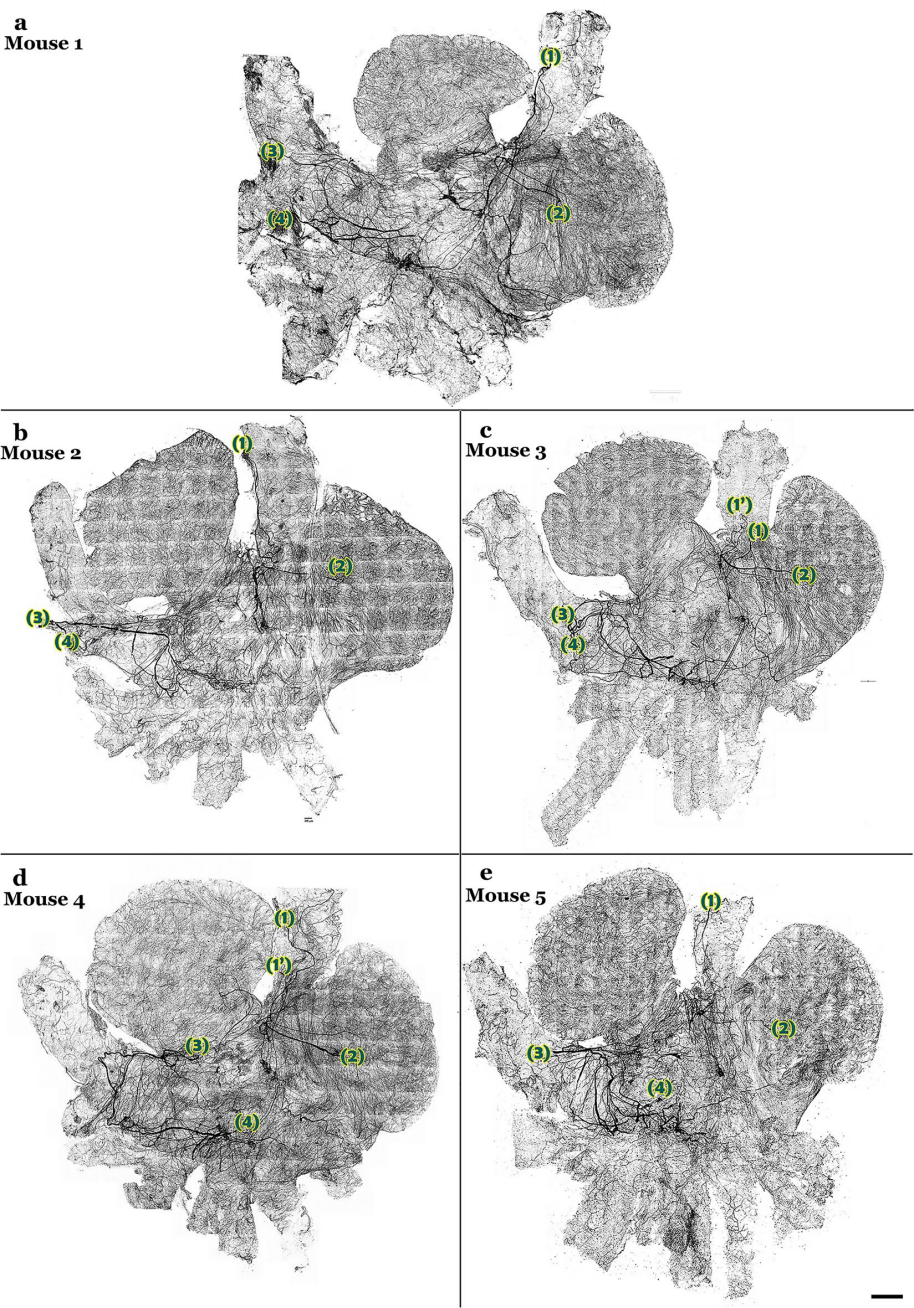
Comparison of TH-IR axon innervation across five animals. Large TH-IR bundles entered the atria mainly through the SVC (bundles 1, 2), LPCV (bundle 3) or the entrance of the PVs to LA (bundle 4). The bundles entering the RA mainly sent their projections to innervate the right atrium, whereas bundles entering the LPCV mainly extended their projections towards the LA. A certain degree of overlap of innervation could be seen in the junction of LA and RA, SA, and AVN. Note: some fibers could have been cut or folded during the dissection which contributed to the few differences across animals. 1’ indicates the additional bundle found in some animals near bundle. Scale bar: 500 μm.

Four major extrinsic TH-IR axon bundles entered the atria (short yellow arrows in Fig. 2), branched into the smaller bundles, and finally ramified into individual axons which covered the entire atria (Fig. 2). Across animals, the number of large TH-IR bundles and their entry locations and innervation fields of the atria were quite consistent. In all atrial tissue preparations, most TH-IR bundles were identified consistently at the medial side of superior vena cava (SVC), entrance of the pulmonary veins (PVs) to the left atrium, and left precaval vein (LPCV) (Fig. 3).

**Figure 4:**
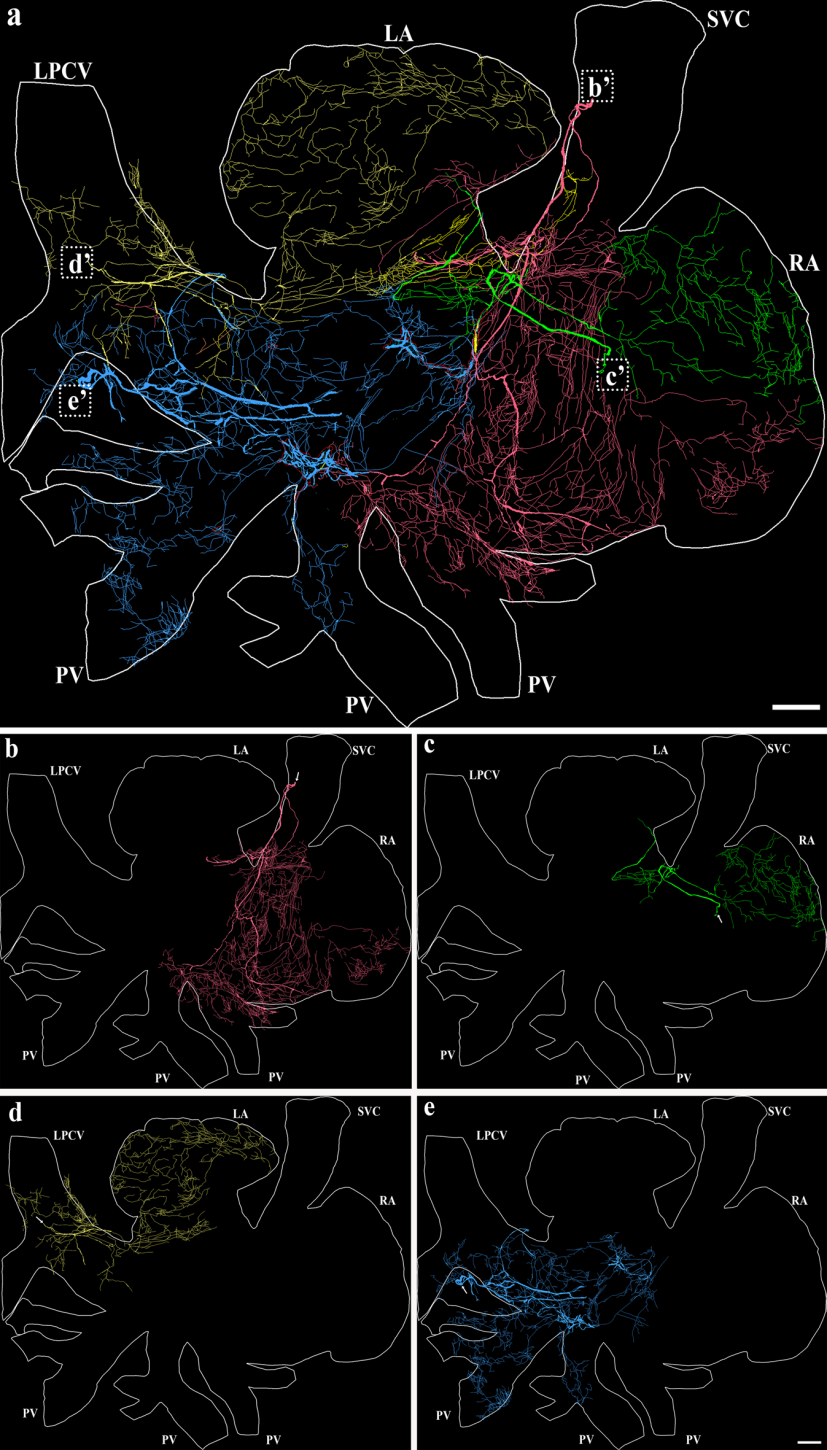
Neurolucida 360 tracing and digitizing of the TH-IR bundles and axons innervating the RA and LA (connected). (**a**) Tracing of TH-IR bundles revealed ~ 4 major bundles entered the atria mainly at the SVC, LPCV or LA-PV junction then bifurcated into small bundles that eventually ramified into individual varicose axons. Two bundles extended the TH-IR axons towards the RA while the other two bundles mainly innervated the LA. These four bundles innervated distinct regions with a certain degree of overlap. (**b**) TH-IR bundle that extended projections mainly towards the base of SVC, SAN, AVN, the lower part of the RAu. (**c**) A TH-IR bundle that extended projections towards the upper part of the RAu, SAN, LA and RA junction, and the medial part of the LA. (**d**) A TH-IR bundle that accessed the LA through the LPCV and mainly innervated the LPCV, the LAu, and the middle of the LA. (**e**) TH-IR bundles entered the LA between LPCV and the left PV to mainly innervate the LPCV, the left and middle PVs, the middle part of the LA and the junction of LA and RA. Arrows indicate the beginning of the parent axon bundle. *RA* right atrium, *LA* left atrium, *LPCV* left precaval vein, *SVC* superior vena cava. Scale bar: 500 μm.

The tracing of TH-IR axons using the Neurolucida system highlighted the trajectory of major bundles effectively. These bundles innervated different regions with a certain degree of overlap (Fig. 4a). TH-IR bundles projected their axons towards the atria via four main topographical pathways:

- ● Bundle 1 entered the atria at the medial side of the SVC and branched into smaller bundles that proceeded towards the SAN, conductive fibers, AVN region, right PV and the lower part of the right auricle (Fig. 4b).
- ● Bundle 2 formed a loop around the origin of the SVC (probably folded during dissection) and sent projections mainly to the upper part of the right auricle and junction of LA and RA (Fig. 4c).
- ● Bundle 3 entered the atria at the LPCV and ramified into individual axons that projected towards the entire left auricle (Fig. 4d).
- ● Bundle 4 entered the atria at the lower edge of the LPCV and projected towards the LA-PV junction, left and middle PVs and junction of LA and RA (Fig. 4e).

Most animals showed a similar trend of TH-IR axon distribution. Some of the variations observed could be due to unintentional folding of bundles during dissection and interindividual variation.

**Figure 5:**
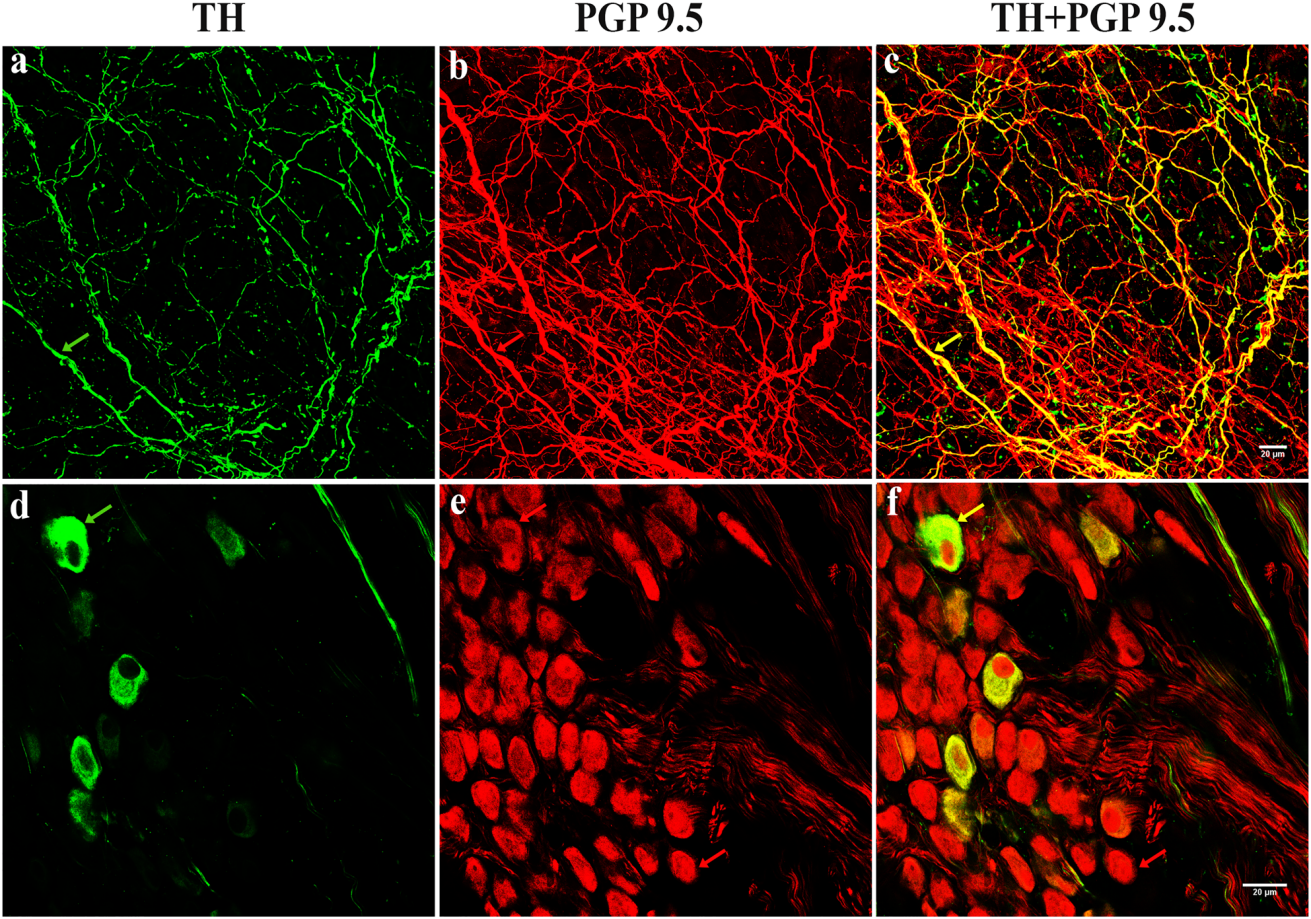
TH-IR and PGP 9.5-IR neurons and axons in the ICG in the atria: Double-labeled. All TH-IR axons and neurons were also positive for PGP 9.5. A few TH-IR neurons were present. *ICG* intrinsic cardiac ganglia, *PGP 9.5* protein gene product 9.5. Arrows indicate the axons and neurons that were dual labeled with TH and PGP 9.5.

To confirm TH-IR axons and neurons were accurately representing neural processes, pan-neuronal marker PGP 9.5 was used. All TH-IR axons and neurons were also PGP 9.5-IR (Fig. 5), indicating that TH-IR fibers (Fig. 5a–c) and neurons (Fig. 5d–f) were indeed neural processes. Additionally, negative controls further confirmed the labeling specificity.

### TH-IR axon innervation of the right and left atrium: density, distribution and morphology

**Figure 6:**
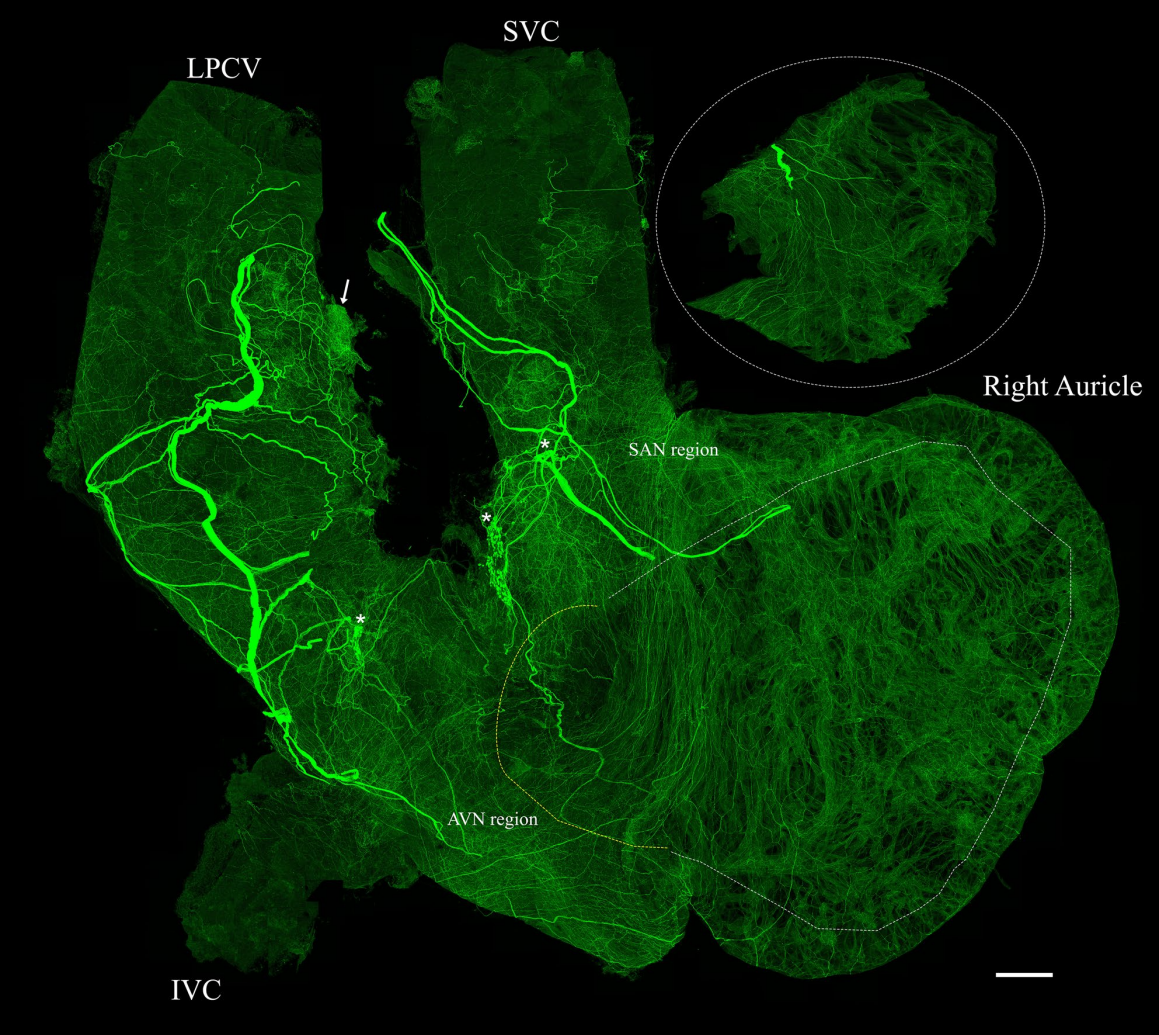
TH-IR axons in the RA of a representative mouse. The RA was separated from the LA and scanned using a 40 × oil objective lens. A montage of ~ 700 maximal projections in the RA showed large TH-IR bundles entered the atrium through the SVC and LPCV, traveled through the cardiac ganglia (asterisks), branched out to small axon bundles, and finally innervated the whole atrium with dense terminal varicosities. Intrinsic cardiac ganglia (asterisks), fat (arrow), white dotted line (boundaries of cut to separate the inner auricle), yellow dotted line (opening of the atrium) (Please zoom in 400 × to view details as well as see the following figures). *RA* right atrium, *LPCV* left precaval vein, *SVC* superior vena cava, *IVC* inferior vena cava, *SAN* sinoatrial node region, *AVN* atrioventricular node region. Scale bar: 500 μm.

**Figure 7:**
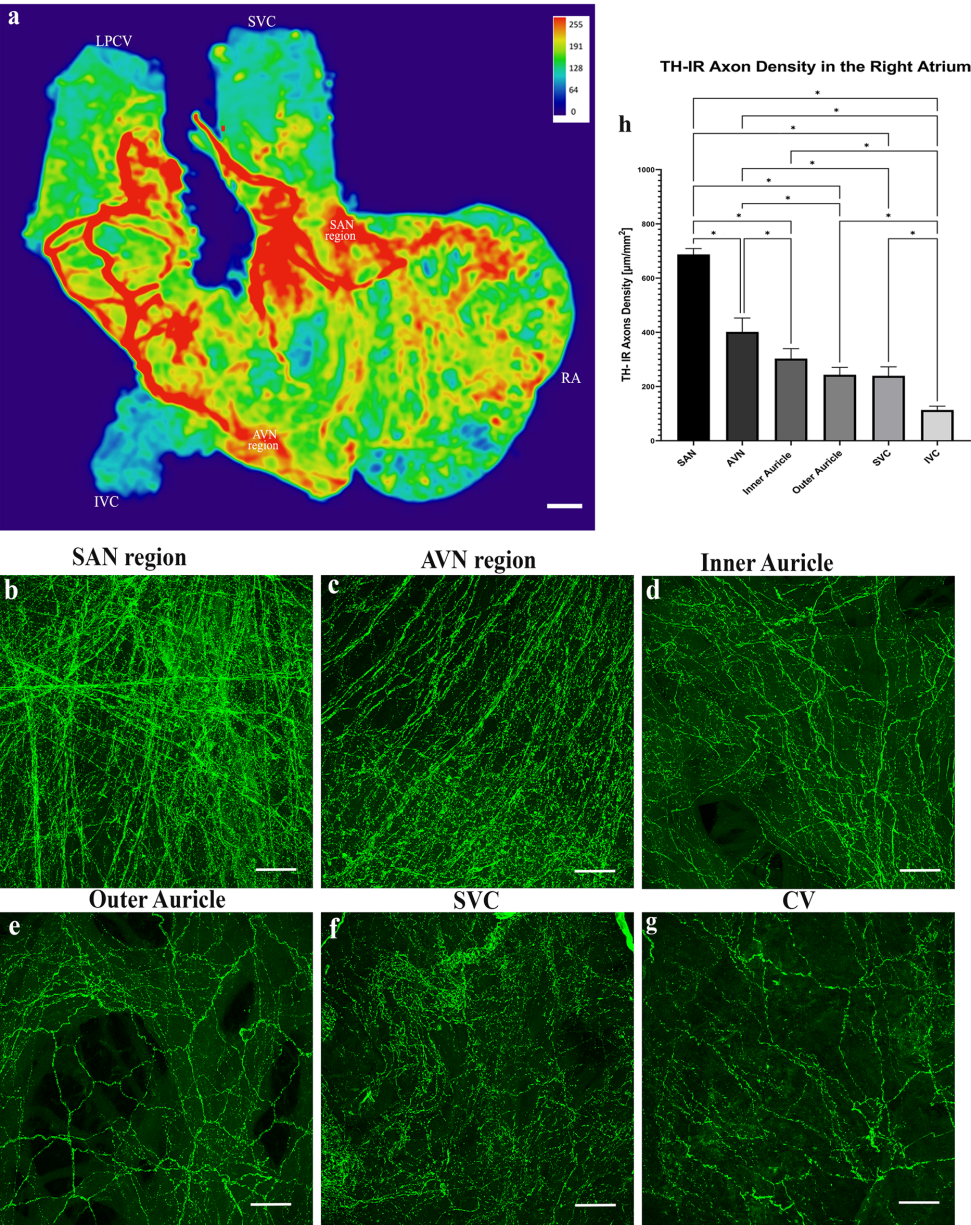
(**a**) Heatmap of the regional density of TH-IR axon innervation in the RA, hot spots are indicated by red color. (**b-g**) Representative images of the regions of interest to show the detailed TH-IR axon innervation in different regions. (**h**) Quantification of TH-IR axons in the RA ROIs of a representative mouse. (Heatmaps were created by Ariege Bizanti with imageJ.1.53t, https://imagej.net/software/fiji/). *P < 0.05. n = 6. Scale bars: 500 μm (in (**a**)); 50 μm (in (**b-g**)).

The distribution of TH-IR axons in the whole right atrium was consistent in all animals**.** A couple of large TH-IR bundles entered the right atrium through the SVC and LPCV (Fig. 6). These large bundles branched into smaller bundles that either passed through the intrinsic cardiac ganglia (ICG) or extended directly to other cardiac targets and ramified into individual axons. The overall density heatmap (Fig. 7a) revealed that TH-IR axon innervation was significantly higher within the region of the SAN compared to other areas (*P* < 0.05, n = 6). The steps for the quantification of TH-IR axon density were delineated in Fig. 1. TH-IR axon density at several regions of interest) in the RA is shown in Fig. 7b–g. The inner and outer walls of the auricles were separated due to their thickness. The density of TH-IR axon innervation in these regions was in the following order from high to low: SAN (687.3 μm/mm^2^ ± 21.63), AVN region (401.7 μm/mm^2^ ± 51.03,), inner auricle (303.1 μm/mm^2^ ± 36.78) and outer auricle (243.4 μm/mm^2^ ± 27.22), SVC (239.5 μm/mm^2^ ± 33.09), IVC (113.6 μm/mm^2^ ± 14.19) (Fig. 7h).

**Figure 8:**
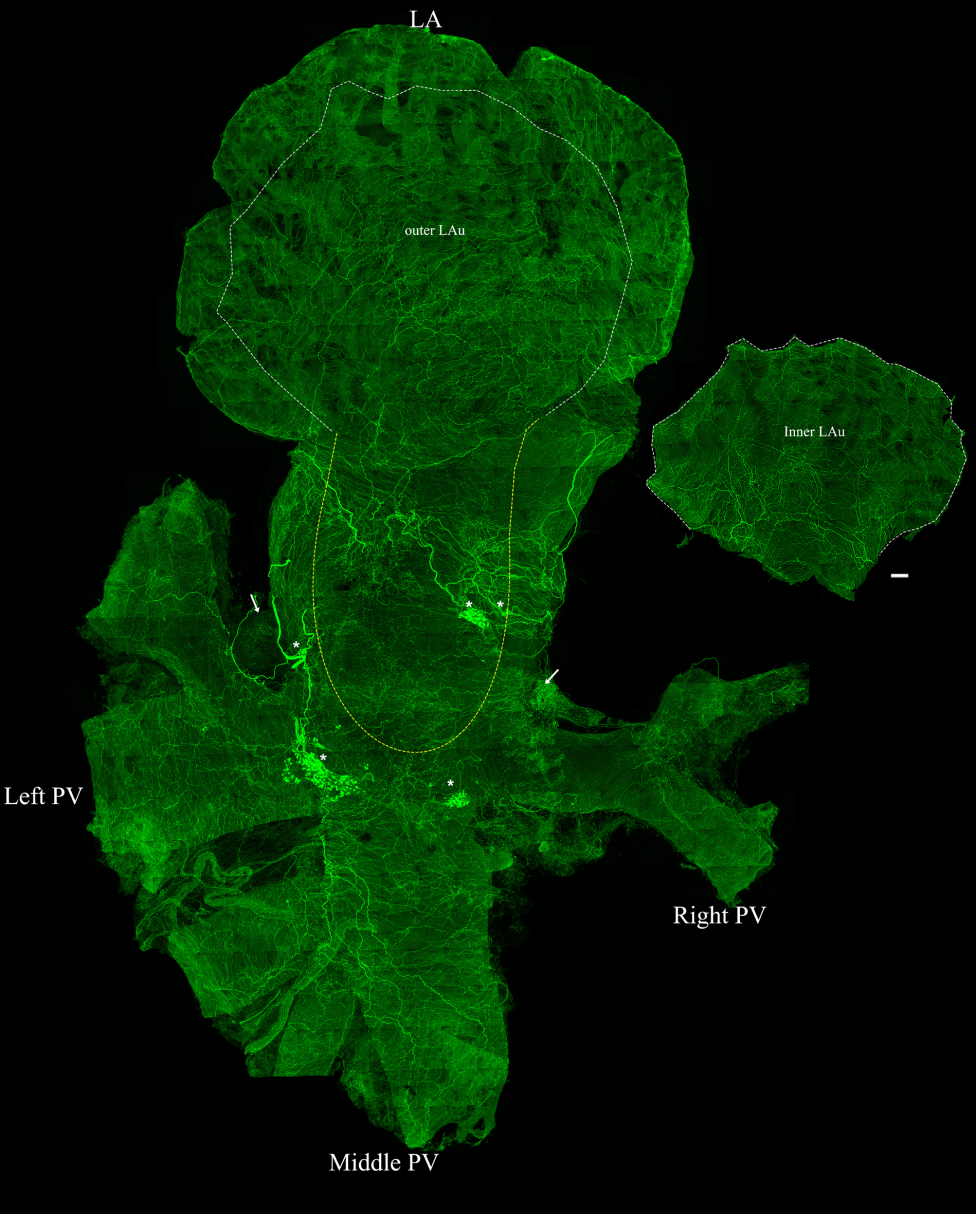
TH-IR axons in the LA of a representative mouse. The LA was separated from the RA of the same representative mouse in Fig. 4. A montage of ~ 700 maximal projections in the LA showed that large TH-IR bundles entered the atrium at the LA-PV junction, traveled through cardiac ganglia, branched out to smaller axon bundles and extended individual axons that innervated the whole atrium with dense terminal varicosities. (Please zoom in 400 × to view details as well as see the following figures). ICG (asterisks), fat (arrow), white dotted line (boundaries of cut to separate the sinner auricle), yellow dotted line (opening of atrium), *LA-PV* left atrium-pulmonary vein junction, *PV* pulmonary vein. Scale bar: 500 μm.

**Figure 9:**
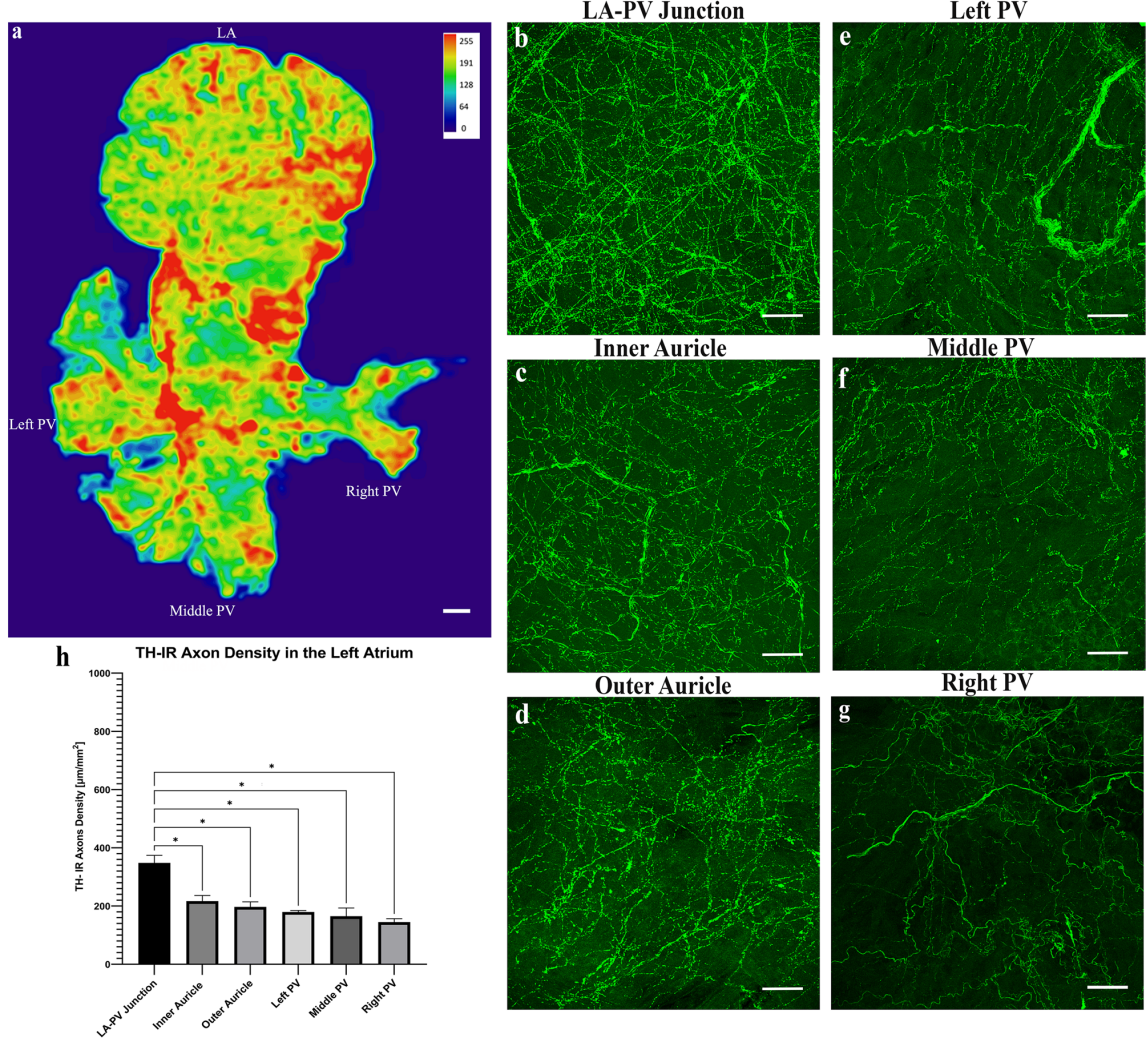
(**a**) Heatmap of the regional density of TH-IR axon innervation in the LA, hot spots are indicated by red color. (**b-g**) Representative images of the regions of interest to show the detailed TH-IR axon innervation in different regions. (**h**) Quantification of TH-IR fibers in LA ROIs of a representative mouse. (Heatmaps were created by Ariege Bizanti with imageJ.1.53t, https://imagej.net/software/fiji/). *P < 0.05. n = 6. Scale bars: 500 μm (in (**a**)); 50 μm (in (**b-g**)).

The distribution of TH-IR bundles and axons in the flat-mount of whole left atrium was determined (Fig. 8). A couple of TH-IR bundles entered the left atrium through the LA-PV junction then bifurcated into smaller bundles. These bundles either extended towards the ICG or directly to other cardiac targets and eventually ramified into numerous axon terminals covering the entire left atrium. This montage clearly showed a holistic view of the sympathetic innervation of the left atrium at single axon/cell/varicosity scale. The overall heatmap of a representative mouse (Fig. 9a) showed the highest density of TH-IR immunoreactivity in the regions of the left atrium within the LA-PV junctions and the roots of pulmonary veins. Regional density analysis of ROIs in the LA (Fig. 9b–g) showed the density of TH-IR axon innervation as following from high to low: LA-PV junction (mean 348.2 μm/mm^2^ ± 26), inner auricle (217 μm/mm^2^ ± 19.17), outer auricle (197 μm/mm^2^ ± 17.42), and pulmonary veins (left PV 179 μm/mm^2^ ± 5.25, middle PV 165 μm/mm^2^ ± 28.44, right PV 144.8 μm/mm^2^ ± 11.85) (Fig. 9h). There was a significantly higher density of TH-IR axons in the middle area of the left atrium represented as LA-PV junction than the auricle or pulmonary vein (*P* < 0.05, n = 6).

A comparison of the TH-IR axon density in the RA and LA showed the highest density of innervation was at the SAN. Of note, TH-IR bundles and ICG were excluded from the density calculations and ROIs selected contained only TH-IR axons to avoid any bias in the quantitative analysis. In the LA, the junction of LA-PVs showed very dense innervation of TH-IR axons in most samples (Fig. 9h). Interestingly, even though the density of TH-IR axons in the PVs were less than that at the LA-PV junction, the axons in the PVs were more continuous and had a more defined pattern. The bundles seen on LA are most likely branches of the large TH-IR bundles on the RA that were dislocated during the separation of RA and LA.

### TH-IR neurons and SIF cells and TH-IR axons in ICG

**Figure 10:**
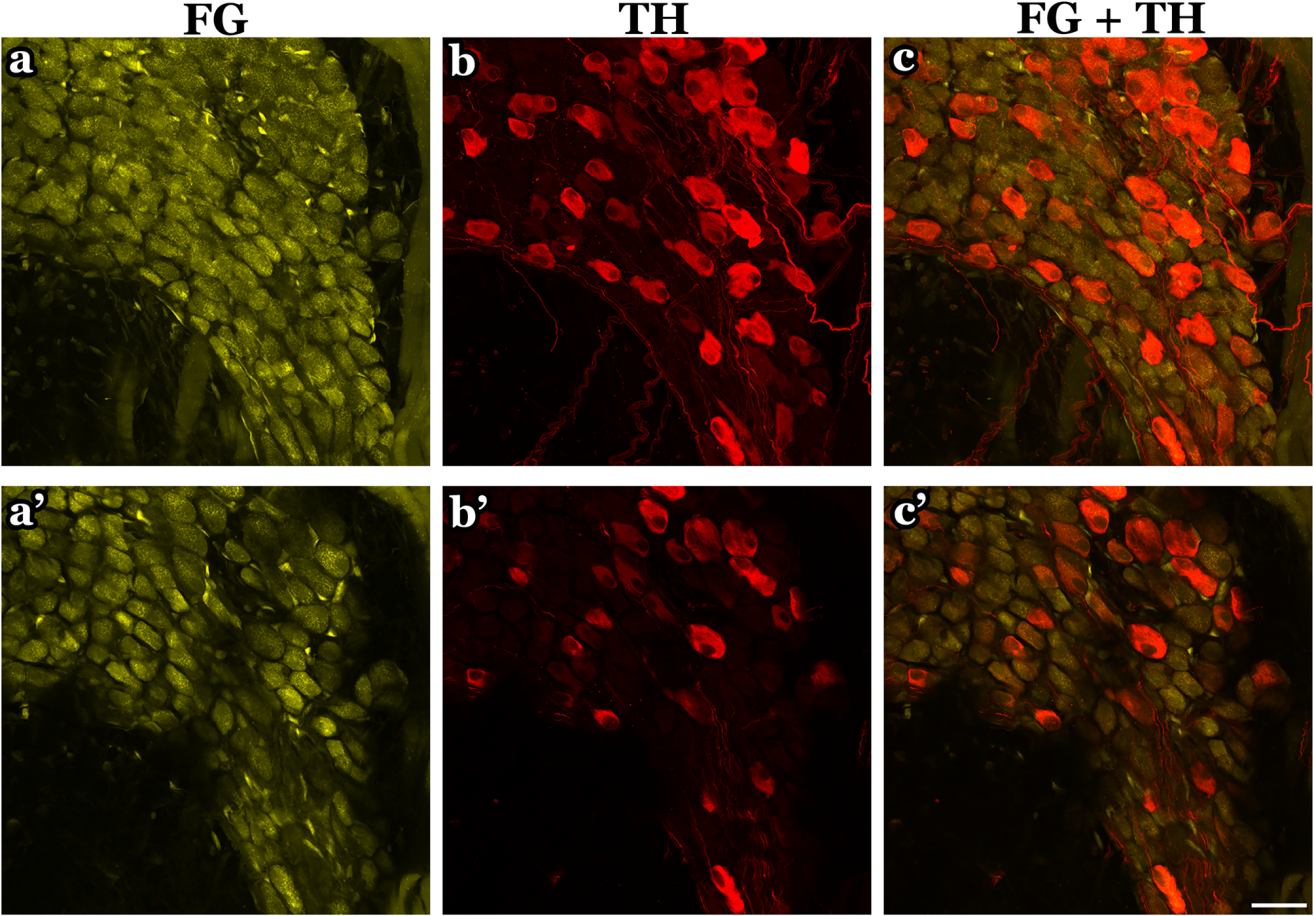
ICG contained a subpopulation of TH-IR neurons. (**a**) Partial projection confocal image of FG-labeled neurons (yellow) in the ICG located on the dorsal surface of the LA. (**b**) TH-IR neurons (red). (**c**) Merged image revealed TH-IR neurons relative to FG-labeled neurons and TH-IR axons passing through the ICG neurons. (**a’-c’**) Single optical sections of the representative images (**a**-**c**). *FG* fluoro-gold, *ICG* intrinsic cardiac ganglia. Scale bar: 50 μm.

**Figure 11:**
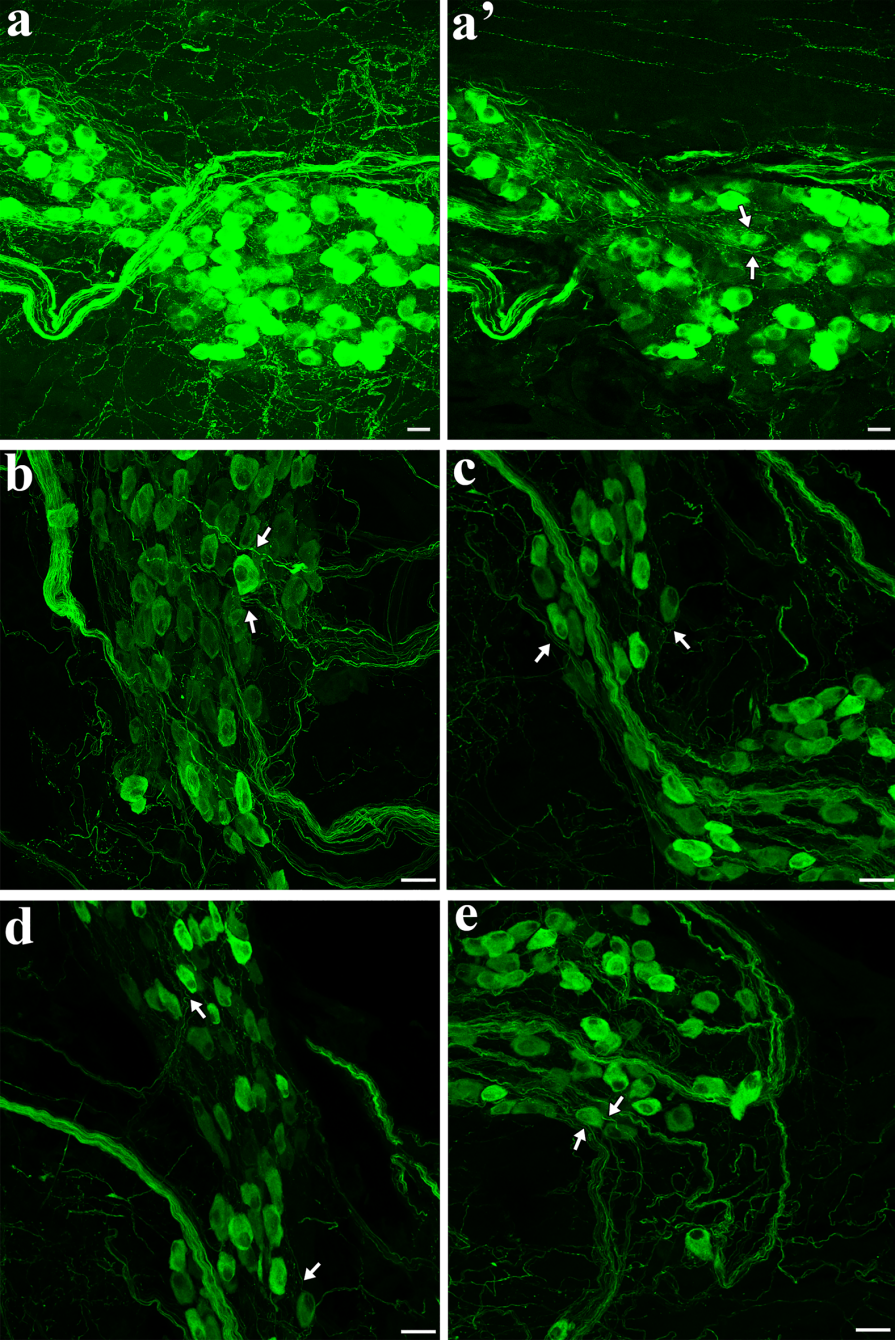
TH-IR axons passed through ICG without apparent innervation. (**a**) Maximal projection image showed TH-IR axons mainly passing through a group of TH-IR neurons. (**a’**) Single optical section of the corresponding image in (**a**). (**b-e**) Partial projection image of different ICG represented the pattern of TH-IR axons passing though rather than directly innervating the TH-IR neurons. Arrows indicate TH-IR axons passing by TH-IR neurons. Zoom 1X (**a,a’**), Zoom 2X (**b-e**). Scale bar: 20 μm.

**Figure 12:**
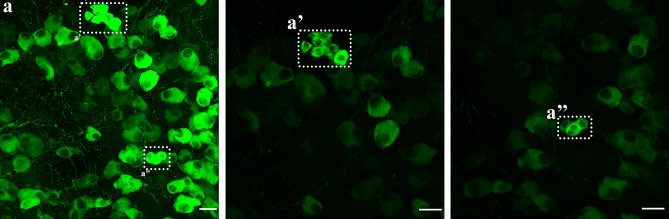
Small intensely fluorescent (SIF) cells in the atria. (**a**) TH-IR SIF cells were grouped into clusters of 3–8 and expressed stronger TH-IR labeling than TH-IR neurons in the ICG. (**a’**,**a’’**) Two optical sections showed two separate SIF cell clusters in the dotted boxes in **a** indicating the intense labeling and small diameter (< 10 μm) of SIF cells. Zoom 1.5X (**a**), Zoom 2X (**a’,a’’**). Scale bar: 20 μm.

In the whole atrial flat-mounts, several intrinsic cardiac ganglia were distributed in the epicardium. The majority of these ganglia were identified near the SAN region, AVN region, and interatrial groove in the connected atria (Fig. 2). When separated, the left atrium had the majority of intrinsic cardiac ganglia in the middle area of the left atrium at the attachment points with the right atrium in the SAN and AVN regions and the entrance of the pulmonary veins (Fig. 8). Some ganglia were also located in the right atrium around the SA region and the epicardial bundles on the LPCV (Fig. 6). ICG were mostly located on the dorsal surface of the mice LA and TH-IR neurons comprised 18–30% of total ICG neurons in maximal intensity projections (Fig. 10a–c) and optical sections(Fig. 10a’–c’). TH-IR fibers were mostly observed passing through the individual ICG (Fig. 11). Even though maximal projection images showed TH-IR axons near the ICG (Fig. 11a), a more detailed evaluation of single optical sections (Fig. 11a’) or partial projections of different ICG (Fig. 11b–e) showed that no TH-IR axon terminals wrapped tightly around the individual ICG neurons. Additionally, small intensely fluorescent cells (SIF) cells were strongly TH-IR (Fig. 12) and were observed in clusters of 3–8 cells, usually dispersed within ICG or near big TH-IR bundles. Optical sections of SIF cells in selected clusters (Fig. 12a’,a”) showed that they have a smaller diameter (< 10 μm) compared to TH-IR neurons in the ICG (~ 20 μm).

### TH-IR axon innervation of vasculature and fat cells

**Figure 13:**
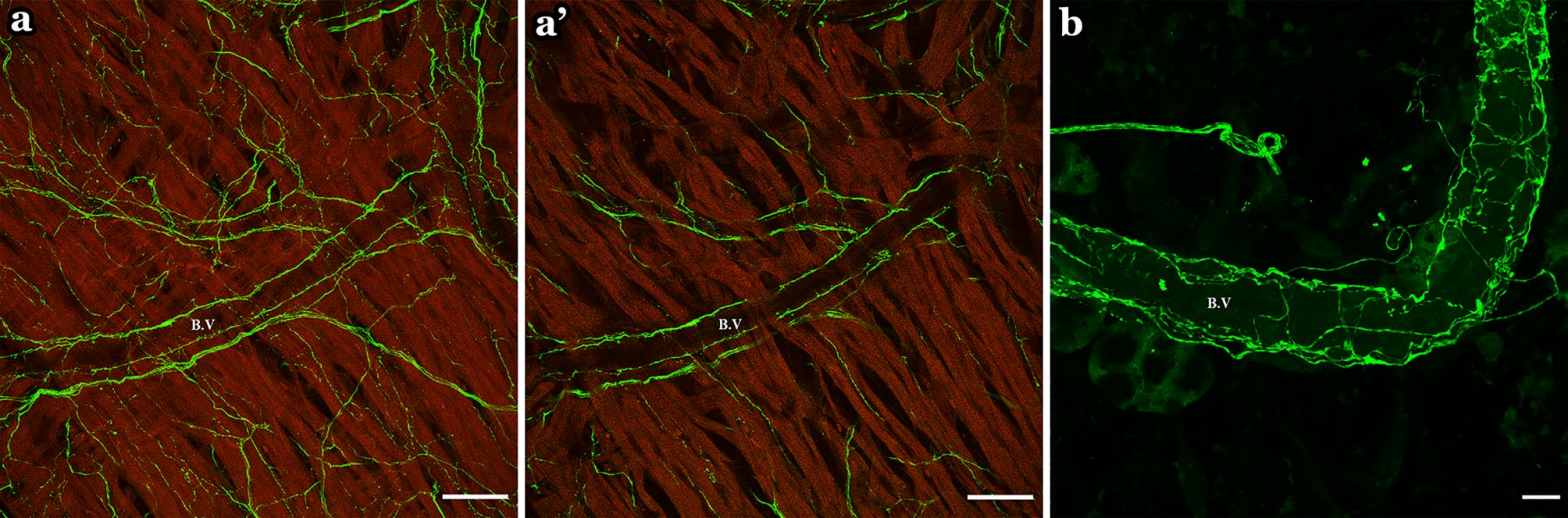
TH-IR axons innervated the blood vessels (B.V). (**a**) Partial projection image showed TH-IR axon innervation along and around the blood vessels. (**a'**) A single optical section of the image in **a** showed TH-IR axons innervating the cardiac muscle. (**b**) showed TH-IR axons innervating the blood vessels. *Red* Autofluorescence . Scale bar: 50 μm (in (**a**,**a’**)) 10 μm (in (**b**)).

In addition to the major veins (SVC, IVC, PVs and LPCV) we identified clearly contoured blood vessels (arterioles) in the left and right atria with TH-IR axons running in parallel to the blood vessel walls or across them (Fig. 13). In the montages, the blood vessels were much less apparent due to the overlays of multiple layers in the maximal projection masking the detailed vascular structures.

**Figure 14:**
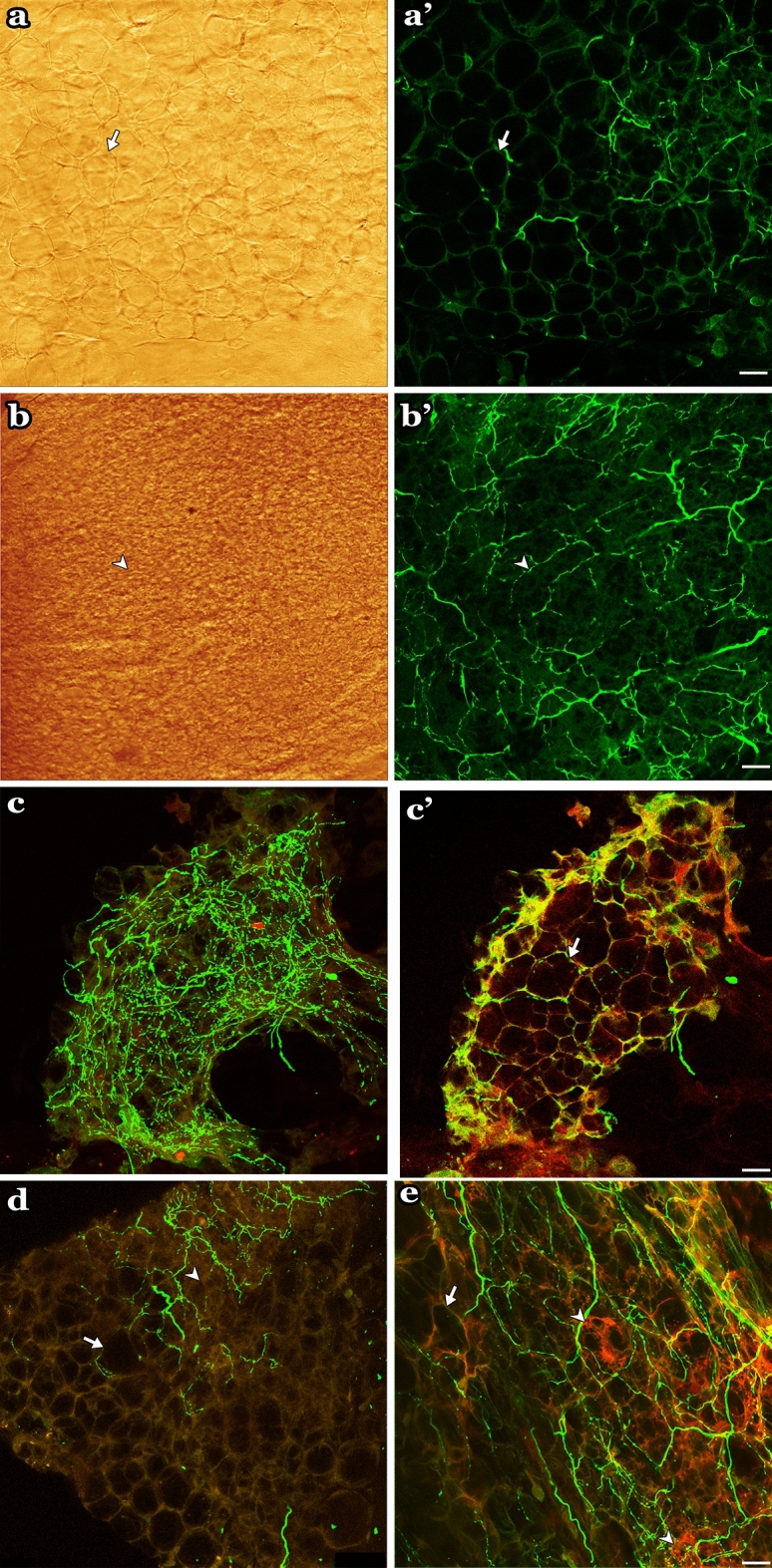
TH-IR axons innervated adipose tissue (white and brown fat cells) (**a**) Single optical section showed WAT morphology using a brightfield channel or (**a’**) fluorescent channel. **b**) Single optical section showed BAT morphology using brightfield or (**b’**) fluorescent channel**.** (**c**) Maximal intensity projection image showed both white and brown adipocytes near the atria being heavily innervated by TH-IR axons. (**c’**) Optical section of the same region in **c** indicating single TH-IR axons running through and around individual adipocytes. (**c-e**). Autofluorescent excitation by UV laser permitted clear visualization of the fat structure, with varicose axons around both WAT and BAT. *Yellow* autofluorescence, *Green* TH-IR.*WAT* white adipose tissue, *BAT* brown adipose tissue. Scale bar: 20 μm.

TH-IR fibers also densely innervated the fat tissues at different layers of the atrial wall. White adipose tissue (WAT) and brown adipose tissue (BAT) were identified by their morphological characteristics using brightfield (Fig. 14a,b) or autofluorescence (Fig. 14d,e). Figure 14c showed TH-IR axons innervated the fat cells in a cluster with numerous varicose terminals. Additionally, the optical sections of the same region showed that TH-IR axons specifically targeted individual adipocytes (Fig. 14c’). TH-IR axon terminals were observed around the boundaries and in between WAT recognized by spherical cells with most of the volume occupied by cytoplasmic lipid droplets and peripherally located nucleus (Fig. 14a’,d). On the other hand, BAT was recognized by multiple vacuoles and darker shade and showed higher innervation by TH-IR axon terminals compared to WAT (Fig. 14b’,e).

## Discussion

Here, we show that several TH-IR axon bundles (presumably sympathetic postganglionic efferent projections) entered the atria from the right and left sides, branched out into individual axons and projected to different fields of the atria with a certain degree of overlap. There was a clear lateralization with the right bundles projecting mainly to the right atrium, whereas the left bundles preferably projected to the left atrium. Asymmetry and regional differences in the cardiac sympathetic distribution were observed in many physiological studies in mice^38^, pig^39^, and humans^40^. Our study provides anatomical evidence for differential regional distribution in mice atria. TH-IR axon bundles were distributed in the epicardium, then bifurcated and formed a terminal network in the myocardium. Moreover, TH-IR axons were observed along/encircling small blood vessels and around WAT and BAT. Regional density analysis showed that the SAN had the highest TH-IR axon innervation. To our knowledge, this work, for the first time, provides a topographical map with quantitative assessment of the TH-IR axon innervation of the mouse whole atria at single cell/axon/varicosity scale.

### Topographical distribution of TH-IR axon innervation in the flat-mount of the whole atria at single cell/axon/varicosity scale

#### Innervation field of TH-IR axons

Several studies have reported the distribution of catecholaminergic nerve fibers utilizing sectioned or whole mounts of partial atrial preparations^16,41–43^. The main limitation of such approaches is that the experimental approach damaged the intricate three-dimensional structures of axons and terminals in these tissues. Additionally, sections or partial flat mounts did not provide a comprehensive topographical map to assess the distribution and morphology of sympathetic postganglionic efferent axons and terminals across the entire atria. Recently, tissue clearing procedures have permitted an enhanced 3D view of the whole heart innervation^22^. However, visibility of fine axons and terminals in the whole heart remained restricted with tissue clearing procedures. In addition, tissue clearance diminished the visibility of other cardiac targets such as ganglion cells, muscles, blood vessels, and adipocytes. In order to highlight the complex patterns of TH-IR axons and their terminal networks in atrial and targets, greater resolution imaging is required.

Our study has addressed these limitations by providing a comprehensive topographical map of the distribution, and morphology of TH-IR axons and terminals in the atria of mice using flat-mounts of the whole atria. Consistent with previous studies on mouse and other species^15,44–46^, we found a very dense TH-IR axon innervation in the atria. Additionally, the entrance points of the major TH-IR bundles to the atria, which were determined in our study, are similar to those that were ascertained previously^14,44,47^. Different from prior reports, our study provided a complete, comprehensive map of TH-IR axons in the atria at single cell/axon/varicosity scale. In the connected atria, we observed that several TH-IR axon bundles (4–5) entered the atria through the SVC and LPCV and bifurcated into smaller bundles that eventually ramified into individual axons forming different projection fields with a certain degree of overlap. Presumably, these bundles were mostly from the left and right sympathetic stellate ganglia. Previous studies using retrograde tracer and stellate ganglionectomy showed that the majority of sympathetic postganglionic innervation originates from the stellate ganglia^48,49^.

Our tracing of TH-IR axons showed clear lateralization as bundles from the right mainly projected towards the right atrium and SAN, while bundles from the left side showed preferential innervation of the left atrium. Our findings reveal detailed regional differences of TH-IR innervation in the entire atria, which enriches our knowledge regarding the differential sympathetic control over distinct regions.

#### Quantitative analysis of TH-IR regional density

Catecholaminergic axon innervation of the atria displays significant anatomical heterogeneity and several studies have attempted to assess the density of cardiac sympathetic nerves at different sites of the heart^50,51^. Although previous studies quantified the density of TH-IR axons at specific sites, they only utilized sections or partial atrial preparations. Thus, a more complete quantitative analysis of TH-IR axon density in the whole heart has not been determined. In our study, we addressed the mentioned shortcomings and analyzed the distribution and density of TH-IR axons in the flat-mount of the whole RA and LA at a high resolution (40X oil lens). The density of TH-IR axons showed regional differences across the atrial wall. In the RA, TH-IR axons and terminals were the densest in the SAN region, followed by the AVN region and other regions, which is similar to what was found in other studies^52–54^. In the LA, the density of TH-IR axons was the highest at the LA-PV junction which was pointed out to be an area richly innervated with sympathetic nerves^55^. The auricles, one of the most prominent structural features of the right and left atrium, play an important role in pumping the blood within the heart with its capacity to expand during each heartbeat^56^. The differential regional distribution of TH-IR axon innervation indicated by our density assessment gives insight to localized effects of catecholaminergic innervation of the atria. Our results could set the foundation for future physiological studies of anatomical remodeling in pathological conditions.

#### TH-IR ICG neurons and TH-IR axons

Traditionally, it was thought that all ICG neurons in guinea pigs and rats were exclusively cholinergic^14,57^. However, recent studies demonstrated that ICG neurons exhibit diverse neurochemical phenotypes (including TH, ChAT, nNOS, VIP, NPY)^58,59^ that extend beyond the traditional concept of cholinergic neurons. A subpopulation of the ICG neurons were also found to be TH-IR in mice which aligns with our findings^10,60^. Similar to previous studies, we have observed the ICG being located primarily on the outer surface of the atria near the entrance of the pulmonary veins to the LA and near the SAN and AVN^25,41^. Our work in mice showed TH-IR neurons in the ICG with TH-IR axons going through the ganglia without apparent innervation. This differs from what was found in guinea pigs and rats where some TH-IR varicosities were seen around ICG neurons^14,61^. These previous findings may be somewhat overestimated by their use of partial preparations that cannot be extrapolated to all ICG neurons. In this study we aimed to assess TH-IR axons that cross through all ICG located on the RA and LA. We found only a few TH-IR axons (if any) were in close contact with ICG neurons. Higher magnification should be used in the future to ensure there is no underestimation of TH-IR axons presence around ICG neurons. In support of this finding in mice, our recent study in pigs showed that TH-IR axons traveled through the ICG without forming varicosities surrounding the principal neurons (PNs)^62^. The lack of TH-IR varicosities wrapping tightly around TH-IR neurons in the ICG contrasts with what was observed in the gastrointestinal tract where TH-IR varicosities tightly surround the PNs in the myenteric ganglia^63^. Prior research indicated that mice ICG are immunoreactive to dopamine-beta-hydroxylase (DBH) and norepinephrine transporter (NET), but they lack vesicular monoamine transporter 2 (VMAT2)^10^. This is in contrast to the nerve fibers and stellate neurons which are positive for DBH, NET, and VMAT2. The lack of VMAT2 renders the neurons in the mice ICG functionally non-noradrenergic due to their inability to transport dopamine and norepinephrine into synaptic vesicles^11^. However, there were limited studies on the function of TH-IR neurons in the ICG, and further studies are needed to explore the functions of TH-IR neurons in the ICG of different species.

### TH-IR innervation of fat cells and vasculature

The sympathetic nervous system plays a crucial role in BAT thermogenesis and WAT lipolysis through its direct innervation of peripheral fat depots^64–68^. Epicardial adipose tissue is an unusual visceral fat depot and has been shown to express its own specific transcriptomic signature^69^. Epicardial fat was described as white adipose tissue with brown-fat-like features^69,70^. We noticed the presence of both types of adipose tissue at multiple locations with predominance of WAT on the atrial epicardium. Similar to our study, recent work that utilized iDISCO tissue clearance, confocal and light sheet microscopy showed a differential density of TH-IR axonal varicosities in BAT and WAT^71^. Further functional studies to investigate the physiological effects of sympathetic innervation of both BAT and WAT in the atria would be highly valuable.

As expected, TH-IR axons were observed in close proximity (running parallel or wrapping around) the vasculature^72^. Identification of the ultrastructure to confirm TH-IR axons formed contacts with the blood vessels using electron microscopy^73^ or physiological studies will be needed. It has been demonstrated that the sympathetic nerves have a major influence on the control of blood flow, blood pressure, and total vascular resistance via its innervation of small arteries^74^. In particular, the sympathetic nervous system has an essential role in maintaining cardiovascular homeostasis and normal physiological activities, including vascular tone and blood pressure.

### Functional implications

Although several studies have described the atrial sympathetic innervation, comprehensive studies that delineate the topographical TH-IR axon innervation of the whole atria and regional differences are currently lacking^40^. Our tracing of the TH-IR axon innervation of the whole atria unraveled the complex axonal network and preferential innervation of distinct regions. The mapping data could be utilized to understand the sympathetic specific control of different regions of the atria and their autonomic responses. In our map, the bundles entering the right side of the atria provided the majority of the sympathetic innervation to the right auricle, right PV, SAN and conductive fibers while the left bundles provided the majority of the sympathetic innervation to the left auricle, interatrial groove (junction of LA and RA) and PVs. Regional and lateral differences in the function of the heart have been indicated previously via the functional studies (mainly in humans) of cardiac sympathetic innervation by the right and left stellate ganglia (SG)^40^. SG block revealed that the right SG is largely responsible for increasing heart rate, slowing atrioventricular conduction, and primarily affects the right atrium as opposed to the left atrium. In contrast, the left SG has a lesser effect on heart rate and atrioventricular conduction and primarily affects the left atrium as opposed to the right atrium^75,76^. Modulating the sympathetic innervation of the atria is becoming an increasingly important therapeutic approach^5^, for example, neuromodulation therapy by electrical stimulation or renal denervation has shown great success in treating diseases like atrial fibrillation via remodeling of stellate ganglion and reducing sympathetic output^77^. Therefore, selective targeting of sympathetic innervation of either side of the heart can have different effects. Our topographical map of TH-IR axon innervation in the atria could be used as a cardiac sympathetic atlas to navigate more precise control of different heart regions.

Knowledge of cardiac sympathetic postganglionic innervation location and density may also help to elucidate the normal physiology and abnormal patterns in certain pathological conditions. Our quantitative analysis shed light onto the atrial regions that received the highest TH-IR axon innervation that could potentially indicate a more precise control in these areas. In the RA we found the highest innervation density of TH-IR axons in the SAN, which supports the fact that the sympathetic nervous system has a role in the fine tuning of heart rate. This could also indicate potential therapeutic targets as blockade of neuronal input with propranolol (beta blocker) leads to a decrease in heart rate^78–80^. In the LA, the highest density of TH-IR axons were observed at the entrance of PV to the LA. The junction of the left atrium and pulmonary veins has been indicated to be a focal source which is responsible for the initiation of atrial fibrillation^81^. Therefore, further functional studies of these great vein-atrial junction regions, which were the most dense with TH-IR axons in our quantitative analysis, are valuable to better understand the physiology and pathology of atrial fibrillation.

Considering that understanding how sympathetic neurons communicate to their cardiac targets is essential for understanding how the heart works^82^, our results provide a basis for understanding the role that TH-IR axons specific innervation play in the control of the normal heart as well as in the diseased heart.

### Limitations

A couple of limitations must be acknowledged:

1. Neurolucida 360 TH-IR axon tracing: Despite our effort to trace TH-IR axon bundles and their projection field, it was not feasible to trace the smallest branches and individual axons in the whole atria. Our continuous collaboration with MBF Bioscience in SPARC MAP-CORE to improve the customized settings for autotracing of our labeled axons should ensure more precise and faster tracing.
2. Density of single or double layers: Due to great differences in thickness of atria in different regions, some areas had to be separated into single layers to ensure fair comparison of the density. Moreover, our regional density analysis of TH-IR axon innervation in the axon was performed using 2D projection images that present the dense structures along the z-axis in a single bidimensional image. To gain a more accurate representation of the innervation considering the depth of the tissue, 3D representation of the entire image stack of the atria should be reconstructed to quantify the density for each image stack.

### Summary and future directions

**Figure 15:**
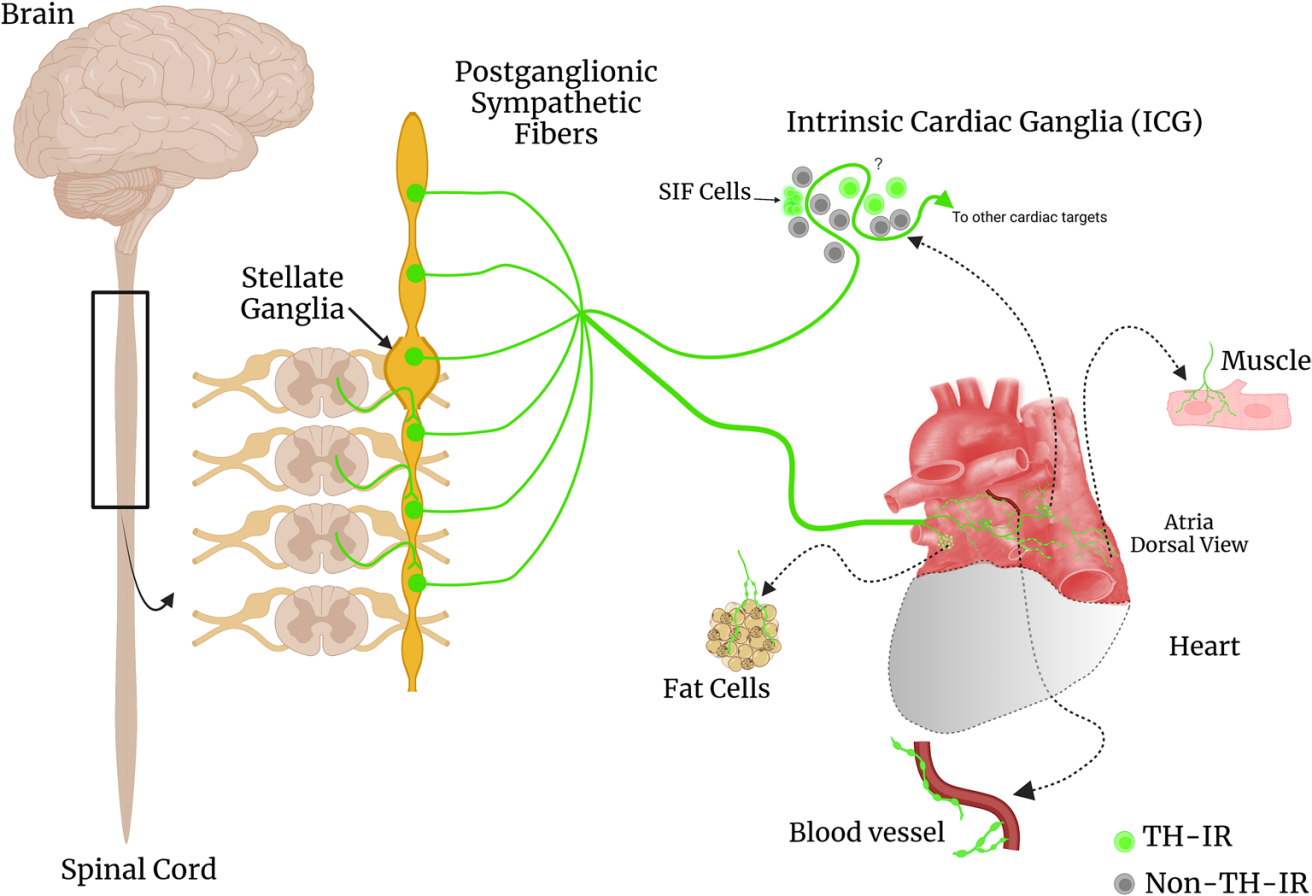
Summary figure describing the main findings of this study. Efferent postganglionic neurons send sympathetic postganglionic fibers to innervate the heart. TH, a commonly used catecholaminergic marker, labeled bundles of nerves, axons, and terminals innervating the atria. Additionally, TH-IR bundles were observed mainly passing through ICG and innervating fat cells and blood vessels. TH-IR neurons comprised about ~ 30% of ICG neurons. The presence of varicosities around TH-IR ICG neurons and whether there is direct innervation remains to be determined in the future (indicated by question mark). *(Created by Ariege Bizanti with Biorender (2022), *https://biorender.com/*).*

We have determined the topographical innervation of TH-IR axons in the flat-mount of the whole atria at single cell/axon/varicosity scale. Several TH-IR axon bundles entered the atria through the SVC and LPCV, and these bundles had different projection fields. A clear lateralization preference was found: the right and left bundles preferably innervated the right and left atrium, respectively. In addition, the regional density analysis showed that TH-IR axon innervation in the RA was more abundant than in the LA. In the RA, The SAN, AVN region and internodal conducting fibers showed higher density than the other regions. LA-PV junction had the densest TH-IR axon innervation in the LA. Furthermore, TH-IR bundles and axons passed through the ICG with very limited innervation around ICG neurons, but densely innervated the blood vessels and fat cells. A schematic diagram that summarizes our main findings is shown in Fig. 15. This work contributes to the cardiac-sympathetic brain connectome. However, anterograde tracer injections into the stellate ganglia to specifically map sympathetic postganglionic projections to the heart should be conducted in the future to address some limitations, including identifying the source of postganglionic TH-IR axons and characterizing terminal structures. In addition, our work provides an anatomical foundation for functional mapping of sympathetic control for the heart as well as evaluation of the remodeling of cardiac sympathetic innervation in chronic disease models (hypertension, diabetes, sleep apnea, heart failure, aging).

## Data Availability

The original high quality figures and data associated with this study^83^, are available through the SPARC Portal (RRID: SCR_017041) under a CC-BY 4.0 license. 10.26275/s2qj-9ggp. Additional data analyzed during the current study is available from the corresponding author upon reasonable request.
